# *In vitro* evaluation of anti-pathogenic surface coating nanofluid, obtained by combining Fe_3_O_4_/C_12_ nanostructures and 2-((4-ethylphenoxy)methyl)-*N*-(substituted-phenylcarbamothioyl)-benzamides

**DOI:** 10.1186/1556-276X-7-513

**Published:** 2012-09-19

**Authors:** Ion Anghel, Carmen Limban, Alexandru Mihai Grumezescu, Alina Georgiana Anghel, Coralia Bleotu, Mariana Carmen Chifiriuc

**Affiliations:** 1 ENT (Otolaryngology), Carol Davila University of Medicine and Pharmacy, Bucharest, 50474, Romania; 2 Department of Pharmaceutical Chemistry, Carol Davila University of Medicine and Pharmacy, Bucharest, 020956, Romania; 3 Department of Science and Engineering of Oxidic Materials and Nanomaterials, Faculty of Applied Chemistry and Materials Science, University Politehnica of Bucharest, Bucharest, 011061, Romania; 4 ENT Clinic, Coltea Hospital, Carol Davila University of Medicine and Pharmacy, Bucharest, 030171, Romania; 5 Stefan Nicolau Institute of Virology, Bucharest, 030304, Romania; 6 Department of Microbiology, Faculty of Biology, University of Bucharest, Bucharest, 060101, Romania

**Keywords:** lauric acid, benzamides, thiourea derivatives, magnetite, anti-biofilm, core/shell nanostructure

## Abstract

In this paper, we report the design of a new nanofluid for anti-pathogenic surface coating. For this purpose, new 2-((4-ethylphenoxy)methyl)-*N*-(substituted-phenylcarbamothioyl)-benzamides were synthesized and used as an adsorption shell for Fe_3_O_4_/C_12_ core/shell nanosized material. The functionalized specimens were tested by *in vitro* assays for their anti-biofilm properties and biocompatibility. The optimized catheter sections showed an improved resistance to *Staphylococcus aureus* ATCC 25923 and *Pseudomonas aeruginosa* ATCC 27853 *in vitro* biofilm development, as demonstrated by the viable cell counts of biofilm-embedded bacterial cells and by scanning electron microscopy examination of the colonized surfaces. The nanofluid proved to be not cytotoxic and did not influence the eukaryotic cell cycle. These results could be of a great interest for the biomedical field, opening new directions for the design of film-coated surfaces with improved anti-biofilm properties.

## Background

In the recent years, the emergence of resistance and multiresistance to antimicrobial substances has led to increasing concerns and interests in finding new antimicrobial agents and identifying new strategies for the treatment of infectious diseases [1-4]. Thiourea derivatives possess many biological activities, including antimicrobial activity, having interesting applications in numerous fields (in agriculture, as ligands useful in coordination chemistry, in analytical chemistry, in anion recognition, and in catalysis).

The synthesis and antibacterial activity of thiourea derivatives has been the subject of numerous investigations. Some new substituted 1,3,5-triazine with 1,2,4-triazole and substituted thiourea and urea were previously synthesized and evaluated for their *in vitro* inhibitory activity against *Staphylococcus aureus*, *Bacillus subtilis*, *Escherichia coli*, *Pseudomonas aeruginosa*, and *Candida albicans*, some of them showing excellent antimicrobial activity [5].

Sarmah and coworkers synthesized and characterized new compounds using the substitution of chlorine in cyanuric chloride by some moieties with biological importance, such as substituted thiourea and heterocyclic systems, in order to achieve enhanced antimicrobial activity against different bacterial and fungal strains, favored by the presence of electron-withdrawing groups on the aromatic ring as compared to compounds with electron-donating groups [6]. The (E)-*N*-[4-(benzamidomethyleneamino)phenylcarbamothioyl]benzamide synthesized by Kurt et al. exhibited *in vitro* antibacterial activity against *B. subtilis*[7].

The biological significance of thioureas and 2-aminobenzothiazoles stimulated research to investigate the synergistic effects of these moieties to afford the design of a new class of heterocyclic thioureas. The new 1-aroyl-3-(substituted-2-benzothiazolyl)-thioureas were found to exhibit moderate to potent activity towards *S. aureus*, *B subtilis*, *P. aeruginosa*, and *E. coli*, as compared to the standard drugs [8]. Some compounds of the novel series of benzothiazolyl thiourea derivatives were equipotent with ampicillin against *S. aureus* and *E. coli* and showed good activity against *Mycobacterium tuberculosis* H37Rv. Also, they were evaluated for *in vitro* cytotoxicity against MCF-7 breast cancer cells [9].

*N*-phenyl- and *N*-benzoylthiourea derivatives obtained by a simple and inexpensive manner display selective antimicrobial activities against *Cladosporium cladosporioides*, *B. subtilis*, and *Micrococcus luteus*. Benzoylthioureas were more active than the corresponding phenyl ones [10].

5-Thiourea oxazolidinones were synthesized and their antibacterial activity against Gram-positive bacteria including methicillin-resistant *S. aureus* and vancomycin-resistant *Enterococcus* was evaluated. This activity was significantly affected by the compounds' lipophilicity, especially the calculated log *p* value [11].

Medical device-related infections account for a substantial morbidity, causing an important economic burden by the increase of antibiotic treatment and hospitalization days, as well as the health-care-associated costs [12-14]. The topological and chemical characteristics of the medical device surface are influencing microbial adherence, the less likely to be colonized being the perfectly smooth, hydrophilic ones. A lot of strategies have been employed to prevent medical device-related infections, one of them being surface modification to prevent microbial population and biofilm formation by (1) the chemical modification of the surface with protein, (2) the modification of the surface with quaternary ammonium salts acting as bacteria-repellent coatings, (3) the incorporation and release of antibiotics from the surface, and (4) the use of noble metals and especially silver on the surface as antimicrobial coatings [15].

There are a lot of reports on the antimicrobial and anti-biofilm properties of different types of nanoparticles, especially metals or metallic oxide-containing ones (silver, copper, gold, and ZnO) [16-26], as well as core/shell nanosystems (e.g., CoFe_2_O_4_/oleic acid, Fe_3_O_4_/oleic acid, and Fe_3_O_4_/PEG_600_) [27-31] that could be manipulated and improved, potentially providing a new method for treating antibiotic-resistant device-related infections [32-35]. In the last year, two articles were published opening a new perspective for obtaining new antimicrobial and anti-biofilm surfaces, based on hybrid functionalized nanostructured biomaterials [36,37]. The first one showed that the *Rosmarinus officinalis* essential oil-coated Fe_3_O_4_/C_18_ strongly inhibited the adherence ability and biofilm development of the *C. albicans* and *Candida tropicalis* tested strains to the catheter surface, and the second showed that the usnic acid-coated Fe_3_O_4_/C_18_ strongly inhibited the adherence ability and biofilm development of the *S. aureus* tested strain to the coverslip surface, as shown by viable cell counts (VCCs) and confocal laser scanning microscopy. These material-based approaches to the control of fungal/microbial adherence could provide both (1) new tools to study mechanisms of fungal/microbial virulence and biofilm formation and (2) approaches to the design of film-coated surfaces or to treat the surfaces of solid and fiber-based materials that prevent or disrupt the formation of fungal/microbial biofilms.

Taking into consideration the aforementioned significant antimicrobial activity of thioureas, in order to continue our work on the evaluation of the bioactivity of compounds with thiourea moiety, we decided to design a new nanosystem combining new 2-((4-ethylphenoxy)methyl)-*N*-(substituted-phenylcarbamothioyl)-benzamides and a Fe_3_O_4_/C_12_ core/shell nanostructure with up to 5-nm size for catheter surface coating, with an improved resistance to *S. aureus* ATCC 25923 and *P. aeruginosa* ATCC 27853 colonization and subsequent *in vitro* biofilm development*.*

## Methods

### Fe_3_O_4_/C_12_: synthesis, characterization, and biological assay

In a previous study [38], we reported the successful synthesis of magnetite coated with lauric acid (C_12_) and its characterization by X-ray diffraction (XRD), transmission electron microscopy (TEM), thermogravimetric analysis, and Fourier transform infrared spectroscopy (FT-IR). The obtained nanopowder was identified as magnetite by XRD. The dimension of the core/shell structure not exceeding 5 nm and its spherical shape were confirmed by TEM analysis. The FT-IR analysis identified the lauric acid on the surface of the magnetite nanoparticles. Treatment for 24 h with Fe_3_O_4_/C_12_ is not cytotoxic on the HEp-2 cell line, this aspect representing an advantage for the *in vivo* use of these nanostructures [38]. Briefly, lauric acid was solubilized in a known volume of distilled-deionized water, corresponding to a 1% (*w*/*w*) solution, under stirring at room temperature. Then, a basic aqueous solution consisting of 28% NH_3_ was added to the lauric acid solution. Thereafter, Fe^2+^ and Fe^3+^ (1:2 molar ratio) were dropped under permanent stirring, leading to the formation of a black precipitate. The core/shell was washed with methanol and separated with a strong NdFeB permanent magnet.

### 2-((4-Ethylphenoxy)methyl)-*N*-(substituted-phenylcarbamothioyl)-benzamides: general synthesis and characterization

**Figure 1:**
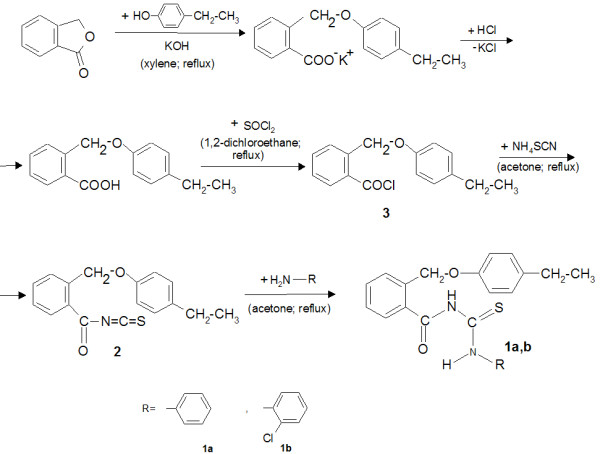
The pathway for the synthesis of the new thioureides (1a, 1b).

2-((4-Ethylphenoxy)methyl)-*N*-(phenylcarbamothioyl)-benzamide (**1a**) and 2-((4-ethylphenoxy)methyl)-*N*-(2-chlorophenylcarbamothioyl)-benzamide (**1b**) were prepared according to procedures described by Limban et al. [39] and according to the scheme plotted in Figure 1.

The new thioureides (**1a**, **1b**) were prepared by heating 2-((4-ethylphenoxy)methyl)benzoyl isothiocyanate (**2**) (obtained from the corresponding chloride (**3**)) with primary aromatic amines in dry acetone under reflux. The acylthioureas are white crystalline solids, insoluble in water, and soluble in acetone and chloroform at normal temperature and in short-chain aliphatic alcohols, benzene, toluene, and xylene at higher temperatures. Their structures were confirmed by elemental analyses, IR, and NMR spectral data.

### Fabrication of coating nanofluid

**Figure 2:**
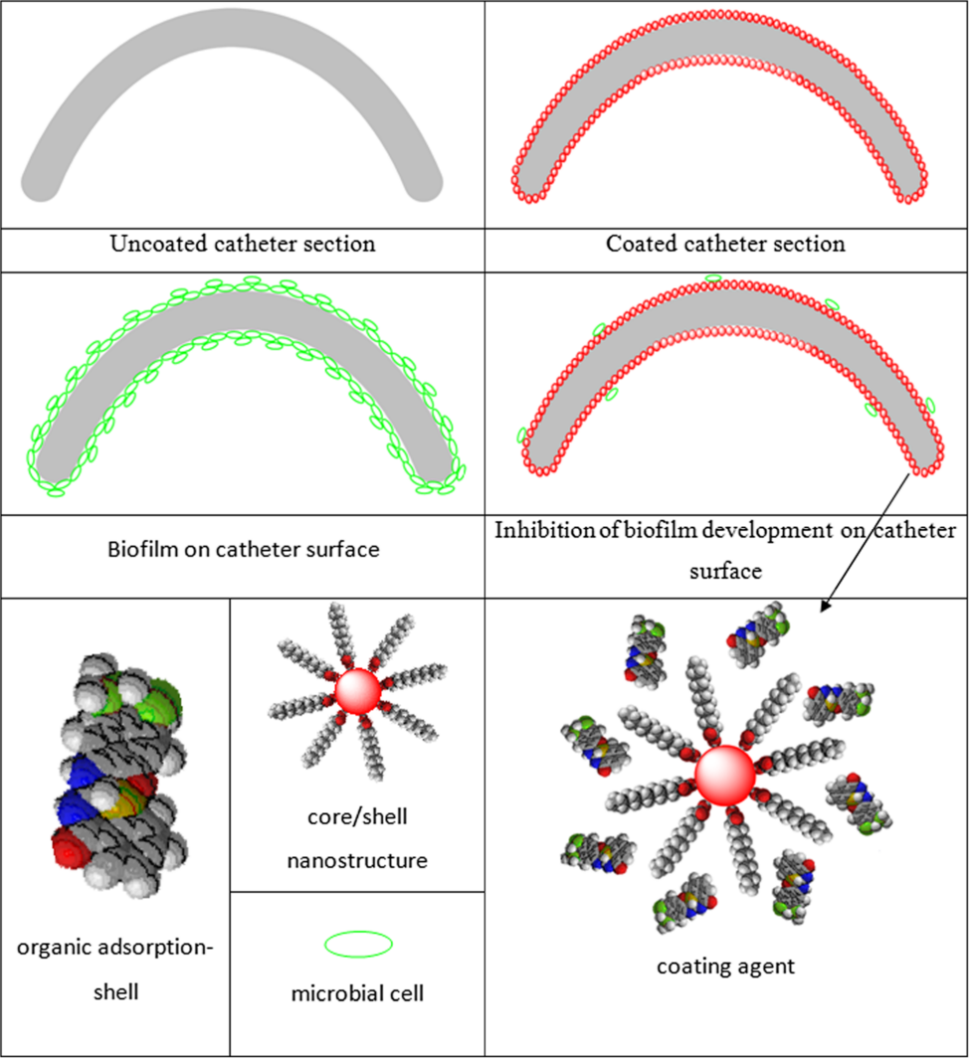
Schematic illustration of biofilm development in the presence/absence of the coating nanofluid.

The adsorption shell represented by 15 mg of (**1a, 1b**) was solubilized in 1 mL of chloroform together with 135 mg Fe_3_O_4_/C_12_ nanopowder. This mix was grounded until complete evaporation of chloroform. This step was repeated thrice in order to obtain a uniform distribution of the organic compounds on the surface of the spherical nanostructure. The fabrication was performed by coating a 1-cm catheter section (the prosthetic device was obtained from a local provider of ENT Coltea Bucharest Hospital) with the nanofluid represented by a suspended core/shell/adsorption shell in CHCl_3_ (0.33% *w*/*v*). The layer of the nanostructured coating on the catheter sections was achieved by submerging the catheter pieces in 5 mL of nanofluid aligned in a magnetic field of 100 kgf applied for 1 s, followed by extemporaneous drying at room temperature. The rapid drying was facilitated by the convenient volatility of chloroform. The coated catheter sections were then sterilized by ultraviolet irradiation for 15 min. Figure 2 presents the schematic illustration of biofilm development in the presence/absence of the coating nanofluid.

### *In vitro* microbial biofilm development to the catheter sections with modified surface

The biofilms were developed using *S. aureus* ATCC 25923 and *P. aeruginosa* ATTC 27853 strains. The microbial adherence ability was investigated in six multiwell plates, in which there have been placed 1-cm catheter pieces with and without nanostructured coating. Plastic wells were filled with a liquid medium, inoculated with 300 μL of 0.5 McFarland microbial suspensions and incubated for 72 h at 30°C. After 24 h, the culture medium was removed, the catheters were washed three times with phosphate-buffered saline (PBS) in order to remove the non-adherent strains, and fresh glucose broth was added. Also, VCCs have been achieved for both working variants (coated and uncoated catheter pieces) at 24, 48, and 72 h in order to assess the biofilm-forming ability of the two strains. The adhered cells have been removed from the catheter sections by vortexing and brief sonication, and serial dilutions ranging from 10^−1^ to 10^−4^ of the obtained inocula have been spotted on Muller-Hinton agar, incubated for 24 h at 30°C and assessed for VCCs.

### Cell cycle analysis

Plastic slides uncoated or coated with the obtained nanofluid were put into wells of 3.5-cm diameter, and thereafter, 3.5 × 10^5^ HCT8 cells and HEp-2 cells were seeded onto the slides and maintained for 24 h at 37°C in 5% CO_2_ and humid condition. The morphology of cells grown on the plastic or coated slides was checked using an inverted microscope after 24 h. For cell cycle analysis, cells were harvested, washed with PBS (pH 7.5), fixed in 70% cold ethanol, and maintained at −20°C overnight. Each sample was washed with PBS, treated with 100 μg/mL RNase A for 15 min, and colored with 10 μg/mL propidium iodide by incubation at 37°C for 1 h. After cell staining with propidium iodide, the acquisition was done using a Coulter Epics XL Flowcytometer (Fullerton, CA, USA). Data were analyzed using FlowJo software (Tree Star, Inc., Ashland, OR, USA) and expressed as fractions of cells in the different cell cycle phases.

### Assessment of cytotoxicity by fluorescent microscopy

HCT8 cells (3.5 × 10^5^) were seeded in each well of a 24-well plate. After 24 h, the cells were treated with 50 μg/mL of the obtained nanofluid. The effect of the nanofluid was evaluated after 24 h by adding 100 μL PI (0.1 mg/mL) and 100 μL fluorescein diacetate. In order to evaluate dead cells (red) and viable ones (green), fluorescence was quantified using a Carl Zeiss Observer.D1 microscope (Oberkochen, Germany).

### Statistical analysis

The statistical significance of the obtained results was analyzed using GraphPad Prism version 5.04 for Windows [40]. We used for comparison the number of colony-forming units per milliliter as revealed by the readings of three values/experimental variants. Logarithmated values were used for statistical analysis. We chose to employ two-way ANOVA and Tukey's multiple comparison tests for revealing significant differences among the analyzed groups.

## Results and discussion

Catheter-related infections continue to be a significant source of morbidity and mortality in patients requiring catheterization [41] and increase medical expenses by prolonging hospitalization. One of the most common etiologies of catheter infections are staphylococci, either coagulase-negative staphylococci or *S. aureus*, and *P. aeruginosa*. There are a lot of studies trying to demonstrate the efficiency of different substances as anti-biofilm-coated agents in reducing the incidence of catheter-associated biofilm infections (i.e., cefazolin, teicoplanin, vancomycin, silver sulfadiazine, chlorhexidine-silver sulfadiazine, minocycline-rifampin, lysostaphin, ciprofloxacin, and protamine sulfate combinations). There are a lot of studies reporting the efficacy of antibiotic-bonded catheters in preventing microbial biofilms from developing. It was demonstrated that the immersion of central venous catheters and arterial catheters in a 50 mg/mL cefazolin solution reduced the catheter colonization with *Staphylococcus epidermidis* from 40% to 2%, proving that antibiotic bonding is an efficient, safe, and cost-effective method of reducing intravascular catheter infections in patients who are in intensive care units [42,43]. Also, other research teams demonstrated that catheter coating with lysostaphin might be more suitable than antimicrobial bonding, due to the rapid coating time of catheters with minimal on-site catheter preparation, and the rapidity of kill would eradicate adherent bacteria within a very short amount of time, eliminating the risk of infections [44]. Nano-silver coatings have been applied to several medical devices, of which catheters, drains, and wound dressings are the most prominent [15].

**Figure 3:**
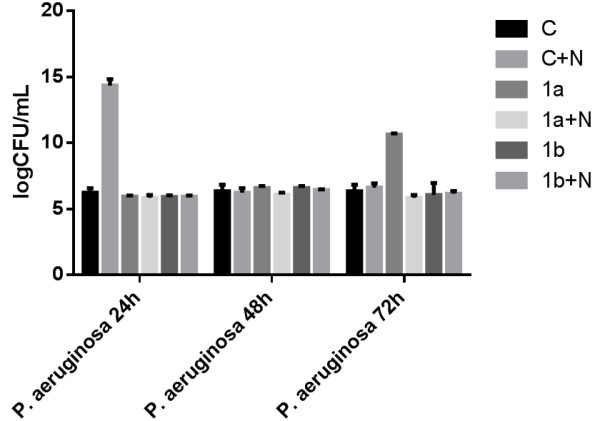
**The VVCs of* P. aeruginosa *cells embedded in biofilms developed on different catheter sections.** C, uncoated catheter; C + N, catheter coated with nanoparticles; 1a, 1b, catheters immersed in the compound solution; 1a + N, 1b + N, catheters coated with N and the chemical compound solution.

Previous studies have demonstrated that the synergism between ciprofloxacin and protamine sulfate significantly enhanced the efficacy of ciprofloxacin against planktonic and biofilm-grown *P. aeruginosa* cells [45]. In our study, concerning *P. aeruginosa* biofilms, catheter coating by nanoparticles alone proved to be significantly more prone to bacterial colonization (*p* < 0.0001) at 24 h than the standard catheter and the catheter sections either immersed in the antimicrobial solution or coated with the nanoparticles loaded with the newly synthesized compounds (Figure 3). The compounds incorporated in nanoparticles (**1a**) and (**1b**) proved to be more efficient than the nanoparticles alone against *P. aeruginosa* biofilm development at 24 h (*p* < 0.0001). It is to be noticed that at 72 h, the compounds incorporated in nanoparticles (**1a**) exhibited a very significant improvement of the anti-biofilm activity as compared with the catheter sections immersed in the soluble compound (**1a**) (*p* < 0.0001).

**Figure 4:**
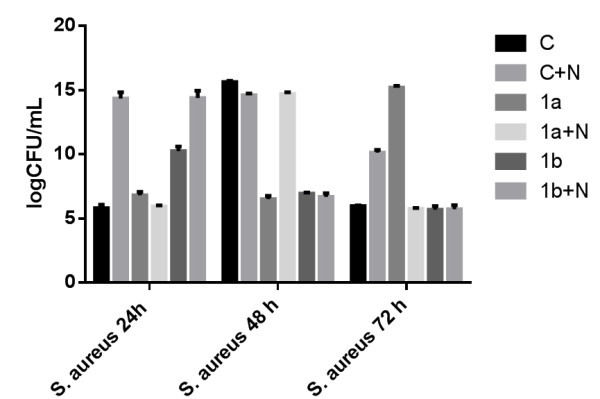
**The VVCs of* S. aureus *cells embedded in biofilms developed on different catheter sections.** C, uncoated catheter; C + N, catheter coated with nanoparticles; 1a, 1b, catheters immersed in the compound solution; 1a + N, 1b + N, catheters coated with N and the chemical compound solution.

Concerning *S. aureus* biofilms, at 24 h, as for the case of *P. aeruginosa* biofilms, the catheters coated only with nanoparticles were significantly more colonized as compared with the uncoated catheter (*p* < 0.0001; Figure 4). However, compound (**1a**) exhibited a strong anti-biofilm activity, the results being very significant when comparing either the uncoated catheter *versus* the catheter immersed in the soluble compound (*p* < 0.0001) or the catheter coated with nanoparticles *versus* the catheter coated with nanoparticles and compound (**1a**) (*p* < 0.0001).

At 48 h, both compounds (**1a**) and (**1b**) in soluble form exhibited protective activity against *S. aureus* mature biofilm development (*p* < 0.0001). Only compound (**1b**) significantly improved the anti-biofilm of the catheter surface in the presence of nanoparticles (*p* < 0.0001). Very statistically significant results have been obtained at 72 h when a strong inhibitory effect of *S. aureus* biofilm development was obtained for the catheter coating constituted of nanoparticles loaded with compounds (**1a**) and (**1b**), as compared with the results obtained for the catheter sections immersed in the compound solution alone or coated only with nanoparticles (*p* < 0.0001).

Our results are demonstrating that the nanoparticle layer alone is not protective against microbial colonization, probably affecting the roughness and the electric charge of the catheter surface, favoring the interaction with the microbial surfaces. On the other hand, our study is clearly proving that the nanoparticle layer is interacting differently with the incorporated substances, influencing their release time in active forms and their antimicrobial activity.

The coating system represented by nanoparticles loaded with (**1a**) proved to be efficient in preventing both the initial formation as well the development of mature microbial biofilms formed by *S. aureus* and *P. aeruginosa*, demonstrating the efficiency of the nanoparticle coating in the delivery of the chemical compound in active forms for a long period of time. These results are also proving that the obtained nanostructured coating agent is not only preventing bacteria to adhere to the catheter surface, but also acting as a biofilm dispersal agent. The molecular mechanisms of bacterial biofilm dispersal are only beginning to be elucidated; however, biofilm dispersal is a promising area of research that may lead to the development of novel agents that inhibit biofilm formation or promote biofilm cell detachment. Such agents may be useful for the prevention and treatment of biofilms in a variety of industrial and clinical settings [46].

**Figure 5:**
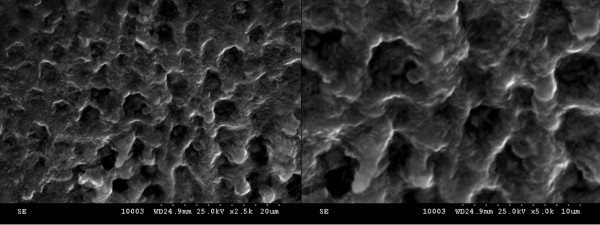
**SEM images showing the uncoated catheter surface colonized with* S. aureus *harvested at 48 h.** The images show a mature biofilm with a rich matrix (left, ×2,500; right, ×5,000).

**Figure 6:**
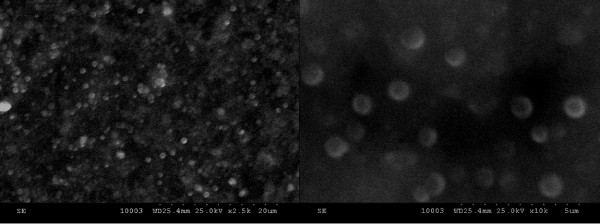
**SEM images showing compound (1b)-coated catheter surface colonized with* S. aureus *harvested at 48 h.** The images show a dense biofilm (left, ×2,500; right, ×10,000).

**Figure 7:**
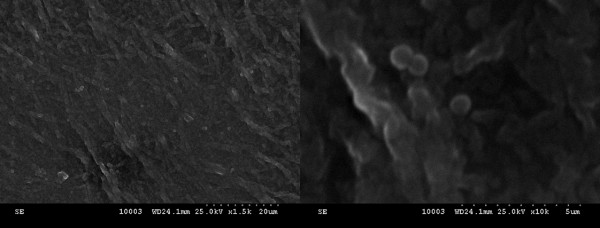
**SEM images showing nanofluid-coated catheter surface colonized with* S. aureus *harvested at 48 h.** The images show a reduced biofilm with rare bacterial cells (left, ×1,500; right, ×10,000).

Scanning electron microscopy (SEM) was used for the evaluation of catheter surface, detection of biofilm, and studying the effect of the coating agents on biofilm development. This technique provides excellent visualization of glycocalyx, which is one of the most prominent features of biofilms and a crucial research subject in searching for alternative antimicrobial and anti-adherent agent treatment [47]. After 24, 48, and 72 h of incubation, the samples were removed from the plastic wells, washed three times with PBS, fixed with cold methanol, and dried before microscopic examination. The samples were visualized using a HITACHI S2600N electron microscope (Chiyoda-ku, Japan), at 25 keV, in primary electron fascicle, on samples covered with a thin silver layer. The culture-based findings were substantiated by the SEM studies of colonized catheter samples, showing the gradual decrease of microbial colonization from the uncoated catheter to the catheter pieces immersed in the compound solution to the catheter pelliculized with nanofluids (nanoparticles and compound solution; Figures 5, 6, and 7).

**Figure 8:**
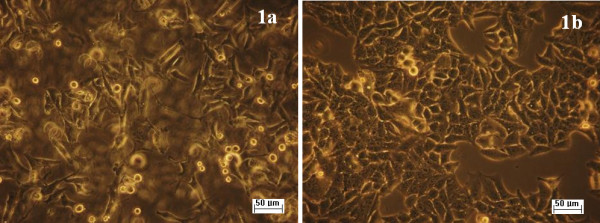
**Inverted microscope images of HEp-2 cells grown on slides treated with the obtained nanofluid.** Phase contrast microscopy, ×200; left, (**1a**); right, (**1b**).

**Figure 9:**
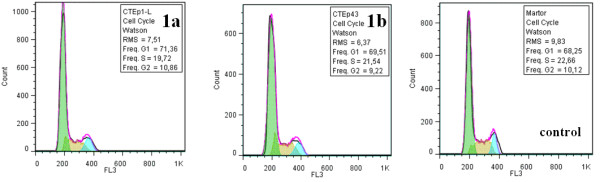
**HCT8 cell cycle analysis after 24-h development on microscopic slides coated with the obtained nanofluid.** From left to right: (**1a**), (**1b**), and control.

**Figure 10:**
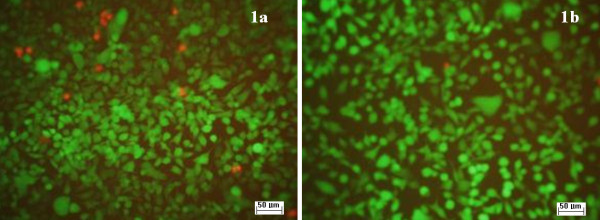
**The effects of the obtained nanofluids on HCT8 cell viability.** IF, ×200; left, (**1a**); right, (**1b**).

When HEp-2 cells were grown on slides coated with the obtained nanofluid, containing nanoparticles and compounds (**1a**) and (**1b**), no changes were observed in their morphology (Figure 8). The analysis of the cell cycle of HEp-2 cells grown on slides coated with nanofluid showed no significant changes of cell cycle phases (Figure 9). Only unsignificant cell death rate was induced by a concentration of 50 μg/mL (Figure 10).

## Conclusions

Our aim was to combine the unique properties of newly synthesized biocompatible Fe_3_O_4_/C_12_ nanoparticles with 2-((4-ethylphenoxy)methyl)-*N*-(substituted-phenylcarbamothioyl)-benzamides in order to obtain functionalized catheter surfaces with improved resistance to *in vitro* microbial colonization and biofilm development. The obtained nanofluids proved to be not cytotoxic and did not influence the eukaryotic cell cycle. The long-lasting efficacy of compound (**1a**) loaded on nanoparticles could be regarded as a future solution to provide persistent, broad-spectrum antibacterial effects with minimal side effects. Taken together, our results could be of a great interest for the biomedical field, opening new directions for the design of film-coated surfaces with improved anti-biofilm properties.

## Competing interests

The authors declare that they have no competing interests.

## Authors’ contributions

IA and AMG participated in the design and coordination of the study. AMG and MCC conceived the study and drafted the manuscript. CL performed the synthesis and characterization of new thiourea. AMG performed the synthesis and characterization of the nanofluid and coated materials. CB and AGA performed the biological studies. All authors read and approved the final manuscript.
